# Development of Lung Cancer Risk Prediction Machine Learning Models for Equitable Learning Health System: Retrospective Study

**DOI:** 10.2196/56590

**Published:** 2024-09-11

**Authors:** Anjun Chen, Erman Wu, Ran Huang, Bairong Shen, Ruobing Han, Jian Wen, Zhiyong Zhang, Qinghua Li

**Affiliations:** 1 School of Public Health Guilin Medical University Guilin China; 2 West China Hospital Chengdu China; 3 Guilin Medical University Guilin China; 4 Department of Neurology Guilin Medical University Affiliated Hospital Guilin, Guangxi China

**Keywords:** lung cancer, risk prediction, early detection, learning health system, LHS, machine learning, ML, artificial intelligence, AI, predictive model

## Abstract

**Background:**

A significant proportion of young at-risk patients and nonsmokers are excluded by the current guidelines for lung cancer (LC) screening, resulting in low-screening adoption. The vision of the US National Academy of Medicine to transform health systems into learning health systems (LHS) holds promise for bringing necessary structural changes to health care, thereby addressing the exclusivity and adoption issues of LC screening.

**Objective:**

This study aims to realize the LHS vision by designing an equitable, machine learning (ML)–enabled LHS unit for LC screening. It focuses on developing an inclusive and practical LC risk prediction model, suitable for initializing the ML-enabled LHS (ML-LHS) unit. This model aims to empower primary physicians in a clinical research network, linking central hospitals and rural clinics, to routinely deliver risk-based screening for enhancing LC early detection in broader populations.

**Methods:**

We created a standardized data set of health factors from 1397 patients with LC and 1448 control patients, all aged 30 years and older, including both smokers and nonsmokers, from a hospital’s electronic medical record system. Initially, a data-centric ML approach was used to create inclusive ML models for risk prediction from all available health factors. Subsequently, a quantitative distribution of LC health factors was used in feature engineering to refine the models into a more practical model with fewer variables.

**Results:**

The initial inclusive 250-variable XGBoost model for LC risk prediction achieved performance metrics of 0.86 recall, 0.90 precision, and 0.89 accuracy. Post feature refinement, a practical 29-variable XGBoost model was developed, displaying performance metrics of 0.80 recall, 0.82 precision, and 0.82 accuracy. This model met the criteria for initializing the ML-LHS unit for risk-based, inclusive LC screening within clinical research networks.

**Conclusions:**

This study designed an innovative ML-LHS unit for a clinical research network, aiming to sustainably provide inclusive LC screening to all at-risk populations. It developed an inclusive and practical XGBoost model from hospital electronic medical record data, capable of initializing such an ML-LHS unit for community and rural clinics. The anticipated deployment of this ML-LHS unit is expected to significantly improve LC-screening rates and early detection among broader populations, including those typically overlooked by existing screening guidelines.

## Introduction

### Lung Cancer–Screening Challenges

Lung cancer (LC) is the second most common cancer and the leading cause of cancer deaths worldwide [1]. It accounted for an estimated 2.2 million new cases and 1.8 million deaths in 2020. Screening for early detection of LC is a crucial strategy to combat this deadly disease [2]. LC-screening guidelines recommend that heavy smokers aged 50-80 years undergo LC screening [3]. Clinical trials have shown about a 20% reduction in LC mortality due to screening with low-dose computed tomography [4].

However, nonsmoking adults and individuals younger than 50 years are often excluded from LC-screening guidelines, despite representing a significant percentage of patients with LC worldwide [5,6]. Statistical risk prediction models, such as PLCOm2012, have been used to recommend LC screening for smokers [7]. The subsequent PLCOall2014 model included nonsmokers in risk evaluation [8], but its impact on screening uptake was unclear. In addition, the adoption of LC screening is low; for instance, only about 5% of the at-risk population in the United States has undergone LC screening [9].

There have been numerous research efforts to overcome these challenges, but their results were inconclusive and unsatisfactory [10]. Researchers have proposed individualized risk-based screening approaches for both smokers and nonsmokers [11]. In 2018, the PLCO model developer reviewed several traditional risk prediction models and suggested that the including biomarkers might help identify individuals who could benefit from LC screening [12]. The PanCan study demonstrated that selecting participants for LC screening based on risk modeling could identify patients with early-stage LC [13]. A recent systematic review concluded that further research is needed to optimize risk-based LC screening [14]. Concurrently, an updated evidence report for the US Preventive Services Task Force indicated that screening high-risk individuals with low-dose computed tomography could reduce LC mortality but might also lead to false positives, resulting in unnecessary tests and invasive procedures [15].

As electronic medical records (EMRs) become prevalent in hospitals, several machine learning (ML) models have been developed using EMR data for LC risk prediction. Kaiser researchers used a small set of preselected variables to identify patients with early-stage LC from routine clinical and laboratory data [16,17]. Stanford researchers developed an ML model to predict the 1-year risk of incident LC using more than 33,000 features from EMR data [18]. Deep learning with convolutional neural networks applied to EMR data from 2 million patients produced a high-performance LC risk prediction model [19]. However, the widespread deployment of these models for risk-based LC screening is yet to be determined.

### The Learning Health System Approach

Over a decade ago, the US National Academy of Medicine (NAM) identified some major shortcomings in the current clinical evidence generation enterprise and proposed the vision of learning health systems (LHS) to address these issues [20-22]. First, many guidelines are primarily based on clinical trials with narrow scopes, failing to fully represent real-world scenarios. For instance, the exclusion of nonsmokers and younger populations from the LC guidelines might be a result of these narrow scopes. Second, the slow dissemination of evidence from discovery to clinical practice contributes to the low adoption rate of LC screening. To address these significant challenges, NAM envisions transforming health systems into LHS to bring necessary structural changes to health care. One of the most significant system-level changes in LHS is that embedding clinical research becomes into routine clinical delivery, facilitating more efficient generation of real-world evidence from real-world data (RWD) of patients and faster dissemination of new evidence to practices. Efficient evidence generation also necessitates innovations in clinical trial methodologies, such as pragmatic clinical trials [23,24].

We believe that NAM’s LHS vision points in the right direction to address the exclusivity, bias, and adoption issues of LC screening. In pursuing sustainable, long-term solutions for inclusive screening and increased screening rates, we believe that system-level innovations are essential. We have focused on two interdependent considerations: (1) more inclusive intervention: exploring data-centric, risk-based LC-screening recommendations instead of blunt exclusions of certain demographic groups; and (2) broader access to the intervention: applying ML-based artificial intelligence (AI) to enable doctors in community and rural primary care to conduct routine risk-based LC screening. Our goal is to assess whether identifying at-risk individuals anywhere using the LHS approach can help close the gap in LC-screening disparities.

These considerations necessitate at least two innovations: (1) a new ML-enabled LHS unit that can continuously improve ML models and thus enhance risk prediction services. Our first ML-enabled LHS (ML-LHS) simulation study using synthetic patient data demonstrated performance improvement of LC risk prediction ML models over time [25]. (2) ML models that are inclusive in terms of patient populations and practical for use in low-resource clinics. Previously, by applying a data-centric EMR ML approach and feature engineering based on a quantitative distribution of health factors derived from EMR data [26]. we successfully developed an inclusive and practical ML model for predicting the risk of nasopharyngeal cancer [27].

### Aims

This study aimed to design an equitable ML-LHS unit for LC screening and to develop an inclusive and practical LC risk prediction model suitable for initializing the LC-screening ML-LHS unit. The future deployment of this new LC ML-LHS unit will aid in implementing risk-based LC screening across populations broader than those currently covered by existing LC-screening guidelines, thereby improving both patient coverage and LC-screening rate.

## Methods

### Hybrid EMR ML Pipeline for Inclusive and Practical LC ML Model

**Figure 1:**
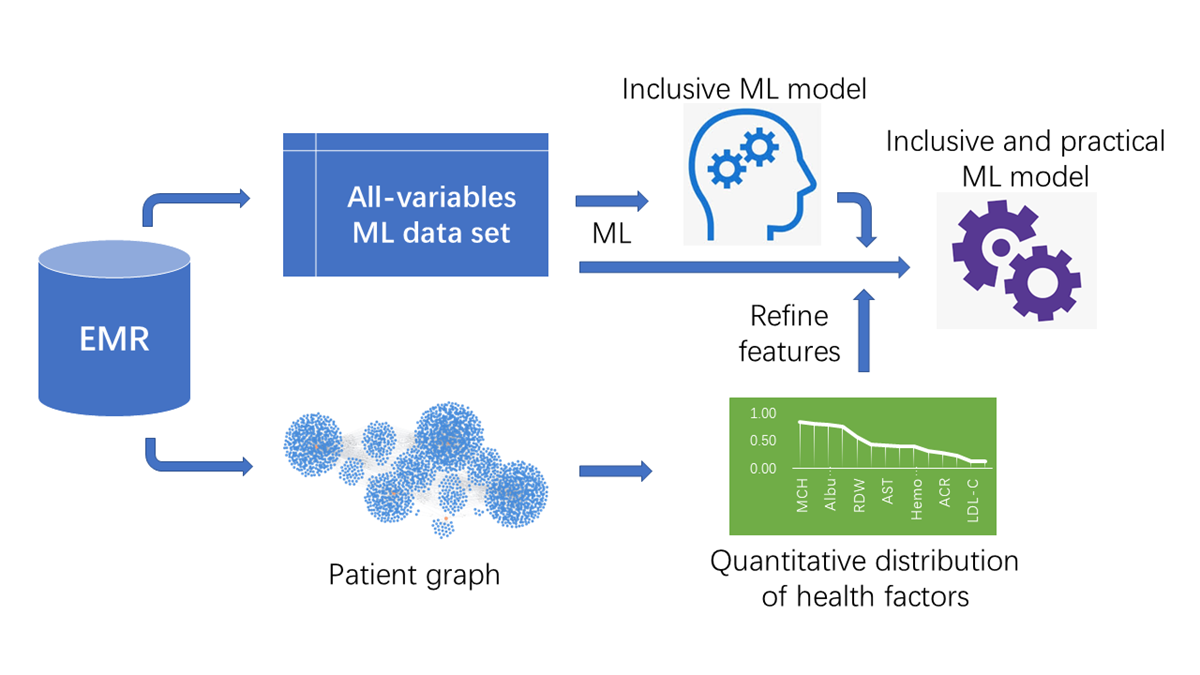
Hybrid EMR ML pipeline for developing inclusive and practical machine learning models for lung cancer risk prediction. The inclusive ML model uses as many health factor variables from EMR as possible. In contrast, the practical ML model uses a small number of variables that are readily available in low-resource clinics. The quantitative distribution of health factor distribution, derived from real-world patient data, aids in refining the features of the inclusive model to formulate the practical model. EMR: electronic medical record; ML: machine learning.

We designed a hybrid EMR ML pipeline to create an inclusive and practical ML model for LC risk prediction (see Figure 1). In step 1, data related to all health factors associated with LC are collected from the EMR. Common ML algorithms, such as XGBoost, are then used to train risk prediction models using these data. In step 2, a patient graph is constructed using all health factors in the EMR, which produces a quantitative LC health factor distribution. In step 3, feature engineering, based on the health factor distribution, refines the model into a more practical one with fewer variables. The recently published patient graph analysis method is used to generate this quantitative distribution of health factors from hospital EMR data [26].

### Standardized Patient Data Collection

Deidentified patient medical records were generated from the hospital’s EMR and relevant databases, covering the period from January 2018 to June 2021. These data sets were securely stored on a data server managed by the hospital’s informatics department. The data set encompassed about 1 million patients and 7 million outpatient and inpatient encounters. The records excluded all fields containing personal information, such as patient names, birth dates, personal IDs, contact details, and addresses. Original hospital identifiers for patients and encounters were replaced by random numbers, not linked to the patients.

Due to the absence of applicable codes for diagnoses in the EMR, Chinese synonyms for LC were used to identify patients with LC. The targeted data set included 1397 patients with LC aged 30 years and older. In addition, 1448 patients aged 30 years and older with no LC were randomly selected to form the background or control data set. We maintained similar numbers of patients in the target and control groups to preserve class balance. However, data standardization, being time-consuming, limited the number of patients in the final structured data set. Based on our experience in building multiple models from EMR, the minimal number is approximately 1000 target patients and 1000 background patients.

Deidentified records of outpatient and inpatient visits, diagnoses, laboratory tests, and procedures were imported into a custom data collection tool on the data server. This tool automatically extracted laboratory test data for storage in a MongoDB database, provided by MongoDB Inc. Our researchers manually curated data from patient record texts and entered them into the database. Data were categorized into 9 categories: disease and condition, symptom, medical history, observation, laboratory test, procedure, medication, treatment, and other risk factors. To overcome the lack of coding and standardization in the records, practical rules were established to ensure consistency in data collection. Synonyms were automatically converted to local “standard terms” with corresponding local codes, culminating in local “standard data.” For each patient with LC, only those data leading to the final diagnosis of LC were collected, forming a patient diagnosis journey (PDJ) object comprising 1 or multiple encounters. For each background patient, all encounters within the 3.5-years period were included. When exporting PDJ data to a comma-separated values file for analysis, only the most recent data for each health factor in the PDJ were selected.

### EMR ML for Inclusive LC Risk Prediction Models

All continuous numeric data in the profiles were converted to categorical data. For example, age ranges were established as 30-50, 50-70, and more than 70 years; drinking levels were categorized as 0-2, and >2 drinks per day; and smoking levels were divided into 0, 1-20, and >20 cigarettes per day. Laboratory test results had predefined categorized such as normal or abnormal, true or false, positive or negative, and high, medium, or low. After this conversion, profiles of patient with LC encompassed more than 58,000 data items and 2066 codes, while background patient profiles comprised more than 46,000 data items and 1298 codes. Subsequently, the profile data were structured into a horizontal table for ML, labeling patients with LC as “1” and background patients as “0.”

Codes were organized based on the number of associated patients with LC. Various sets of codes, exceeding a cutoff of 10 patients with LC, were selected by different criteria for ML. For the LC risk prediction study, all codes related to cancer diseases, procedures, medications, and treatments were omitted. In addition, diagnostic imaging procedures commonly used for patients with cancer but not for background patients were also excluded.

In developing ML models, we used the XGBoost Python library [28]. XGBoost is known for parallel tree boosting and its efficient management of missing data. The Python library scikit-learn from Scikit-learn.org was used for all other ML tasks [29]. The free Jupyter Notebook tool was used to conduct ML experiments [30]. The Pandas library was used for reading and writing comma-separated values files and manipulating data tables. The data set was divided into training (60%), tunning (20%), and validating (20%) subsets. Using the default hyperparameters, the XGBoost classifier was fitted with the training and tunning sets, and the resulting model was independently validated by the validation data set [31]. The model’s effectiveness in risk prediction was evaluated using key metrics such as recall, precision, area under the receiver operating characteristic curve (AUROC), and accuracy. Receiver operating characteristic (ROC) curve and reliability (or calibration) curve were drawn by calling the corresponding Scikit-learn functions.

By comparing the performances of models built from different variable sets, an inclusive variable set was established. Using this set, XGBoost was compared with 3 other commonly used algorithms: random forest (RF), support vector machines (SVM), and k-nearest neighbors (KNN). These algorithms were executed using Scikit-learn classifiers with default parameters. The main reason for evaluating only the common algorithms is because they are promising in delivering the initial acceptable performance required by our LHS design, and their deployment is easier and cost-efficient. Only if this test fails will we test more complex algorithms like neurol networks.

### Building Practical ML Prediction Models

In the final refinement step of our hybrid ML pipeline, a quantitative distribution of LC health factors was generated directly from the same EMR data through patient graph analysis [32]. In the patient graph, health factors are connected to patients with LC and background patients with no LC. The difference in the number of connections to patients with LC versus patients with no LC, called the “connection delta ratio” (CDR), was calculated for each health factor. Sorting the health factors by CDR in descending order provided a quantitative distribution of the health factors. Most of the top health factors with a CDR above a threshold were verified as LC risk factors or were correlated with LC in a literature review. This distribution laid the groundwork for grouping risk factors, selecting only 1 representative factor from each group for the ML model. For instance, pains at different body sites were combined into a single “pain” factor. Data for each variable group were also consolidated, considering the representative variable for the group as true if any of the variables in the group was true.

The following criteria were applied to select a small number of variables for the practical variable set: (1) ensuring that the number of essential variables remained fewer than 30 while achieving key prediction performance metrics (recall, precision, and accuracy) above 80%; (2) using consolidated variables based on the risk factor distribution wherever feasible; (3) minimizing the number of required laboratory tests; and (4) using imaging observations obtainable through simple chest radiographs. The rationale for these empirical criteria is to make the deployment and adoption of the model more practical in low-resource clinical settings, where data for only a small number of variables may be available. However, the LHS starting model should strike a balance between a minimal number of variables and acceptable performance metrics. We tested and compared feature selections using XGBoost. After determining a practical set, we ran RF, SVM, and KNN algorithms for comparison. All models were trained and evaluated using the default parameters of the classifiers. The XGBoost base model used the following default hyperparameters: scale_pos_weight = 1, n_estimators = 500, max_depth = 6, eta = 0.3, gamma = 0, reg_lambda = 1.0, early_stopping_rounds = 5, and eval_metric = 'logloss'.

### Ethical Considerations

This retrospective study of EMR patient data received approval from the Institutional Review Board of Guilin Medical University Affiliated Hospital (number QTLL202139). Prior to data usage, our research team underwent training in patient data security and privacy policy of the hospital.

## Results

### Design of ML-LHS Unit for LC Screening

To improve patient inclusivity and adoption in LC screening, we designed a novel ML-enabled LHS unit for LC screening within a clinical research network (CRN). The CRN is led by a central hospital and participated by numerous clinics in surrounding communities and rural areas. The central hospital is tasked with developing an inclusive and practical LC risk prediction ML model to initialize the LHS unit and providing an AI tool online for clinic use. Primary physicians in these clinics are responsible for routinely using the AI tool to assess LC risk in all patient populations in the CRN. At-risk patients are recommended for LC screening. The hospital also continuously updates models with new patient data, validates models, and deploys improved models for predictive services.

### Inclusive LC Risk Prediction ML Models

A total of 2845 patients, comprising 1397 patients with LC and 1448 patients with no LC, were selected from the EMR of a Chinese hospital. The cohort consisted of 60.8% (1731/2845) men and 39.2% (1114/2845) women. Agewise, 19.6% (557/2845) patients were between 30 and 50 years of age, 58.1% (1654/2845) were between 50 and 70 years of age, and 22.0% (625/2845) were older than 70 years. Within the patient group with LC, 19.8% (277/2845) had a history of smoking, while 80.2% (1120/2845) did not. Since the data set includes a significant number of patients outside the typical LC-screening guideline–recommended demographic, which usually targets heavy smokers aged 50-80 years, the resulting LC risk prediction models were more inclusive, encompassing a broader patient population aged 30 years and older, regardless of smoking status.

**Figure 2:**
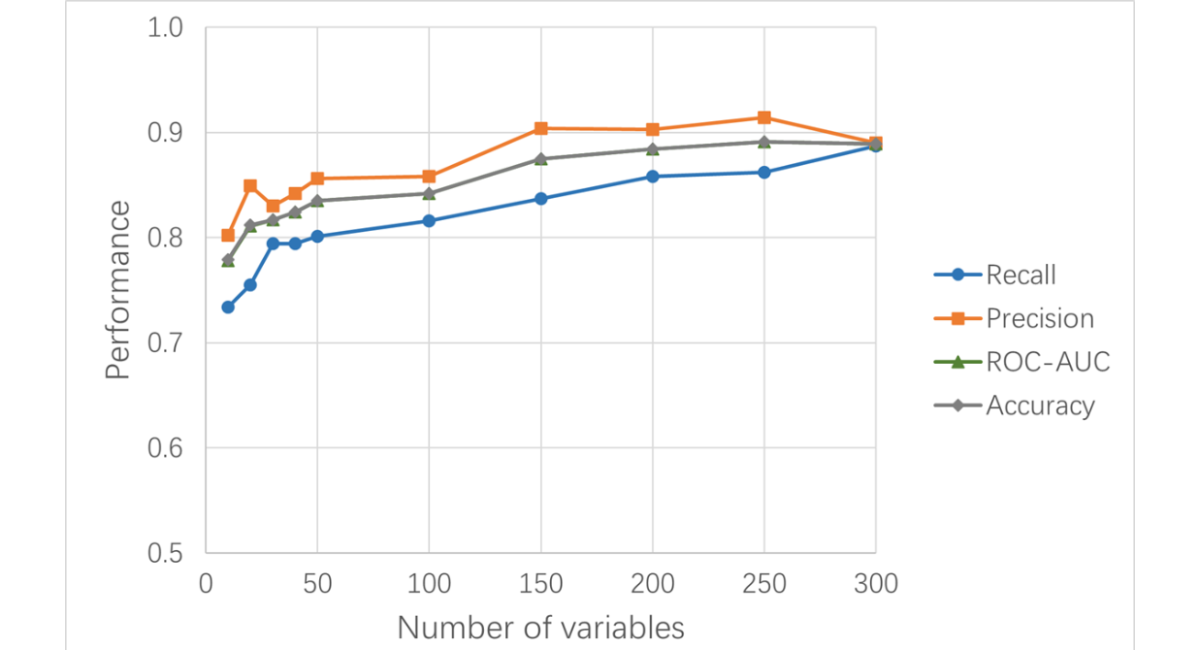
Trends in performance metrics of XGBoost lung cancer risk prediction models with varying numbers of variables. Base models were trained using default settings. ROC-AUC: area under the receiver operating characteristic curve.

To develop an LC risk prediction XGBoost model with default settings, we compared different sets of top-ranked health factors (including diseases, symptoms, medical histories, laboratory tests, observations, and other risk factors) from a list of more than 2000 factors, sorted by each factor’s prevalence in patients with LC. As the number of variables exceeded 200, key model performance metrics plateaued, reaching 0.85 for recall, 0.90 for precision, 0.88 for AUROC, and 0.88 for accuracy (Table 1 and Figure 2). Consequently, a set of 250 variables was selected as the inclusive variable set (denoted as “iv250”).

**Figure 3:**
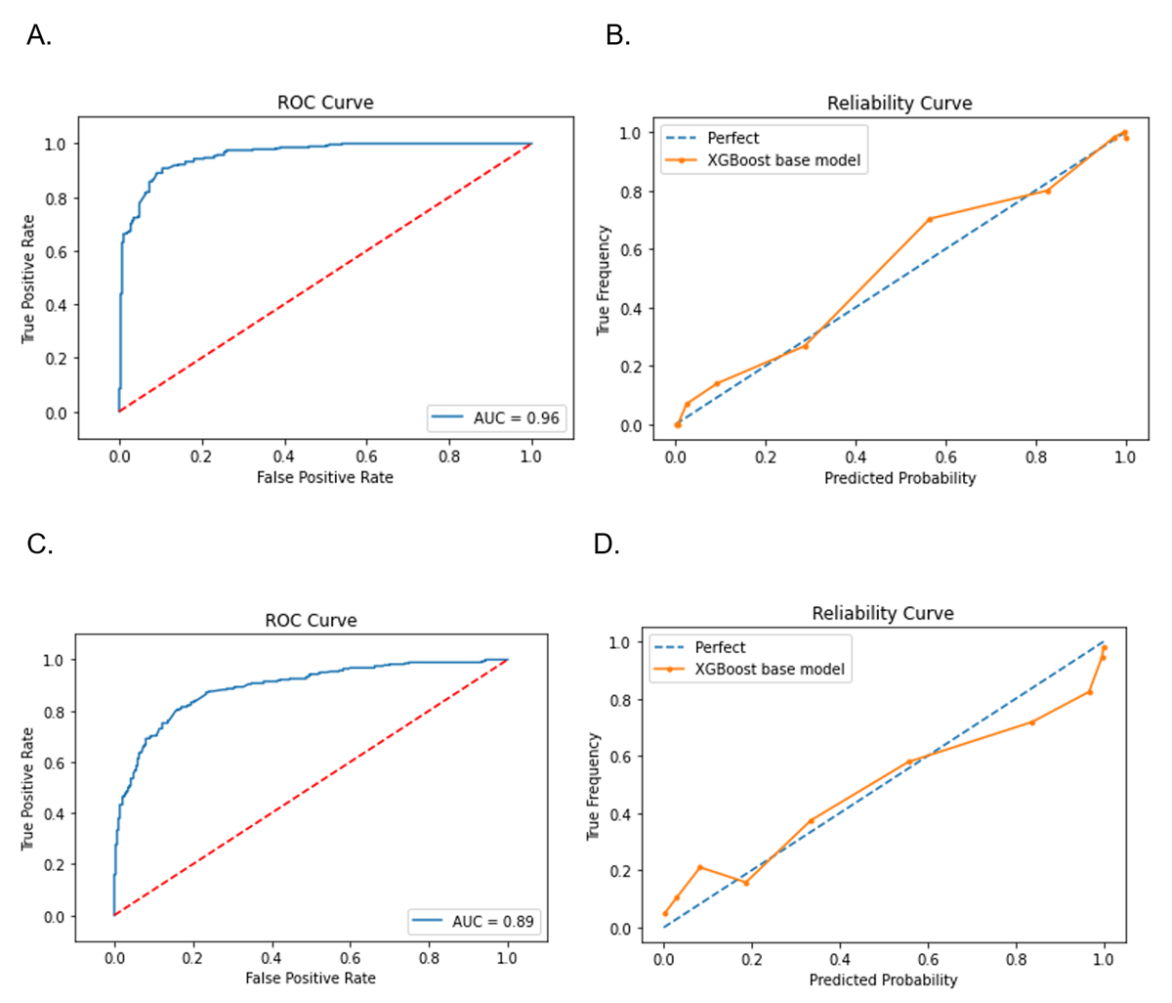
ROC and reliability curves of XGBoost models for lung cancer risk prediction. Models were trained with the default settings. (A) ROC curve for the inclusive model using 250 variables (iv250). (B) Reliability curve for iv250. (C) ROC curve for the practical model using 29 variables (pv29). (D) Reliability curve for pv29. ROC: receiver operating characteristic.

Using the iv250 set and default parameters, we compared XGBoost with other common algorithms such as RF, SVM, and KNN. Table 2 demonstrates that XGBoost and SVM achieved similarly high performance levels, with 0.86 for recall, 0.90 for precision, 0.89 for AUROC, and 0.89 for accuracy. The ROC curve and the reliability curve of the iv250 XGBoost model are shown in Figure 3.

**Table 1:** Performance metrics of the XGBoost lung cancer risk prediction models with different numbers of variables.

Metrics^a^	Number of variables									
	10	20	30	40	50	100	150	200	250	300
Recall	0.734	0.755	0.794	0.794	0.801	0.816	0.837	0.858	0.862	0.887
Precision	0.802	0.849	0.830	0.842	0.856	0.858	0.904	0.903	0.914	0.890
AUROC^b^	0.778	0.811	0.817	0.824	0.835	0.842	0.875	0.884	0.891	0.889
Accuracy	0.779	0.812	0.817	0.824	0.835	0.842	0.875	0.884	0.891	0.889

^a^The XGBoost machine learning base models were configured with default settings.

^b^AUROC: area under the receiver operating characteristic curve.

**Table 2:** Comparison of machine learning model performance using different algorithms for lung cancer risk prediction with default parameters^a^.

Algorithm		XGBoost	Random forest	Support vector machines	K-nearest neighbors
**The inclusive 250-variable set (iv250)**					
	Recall	0.862	0.872	0.887	0.667
	Precision	0.914	0.875	0.909	0.715
	AUROC^b^	0.891	0.875	0.900	0.703
	Accuracy	0.891	0.875	0.900	0.703
**The inclusive and practical 29-variable set (pv29)**					
	Recall	0.805	0.816	0.748	0.649
	Precision	0.825	0.830	0.858	0.832
	AUROC	0.819	0.826	0.813	0.760
	Accuracy	0.819	0.826	0.814	0.761

^a^All machine learning base models used default settings.

^b^AUROC: area under the receiver operating characteristic curve.

### Practical LC Risk Prediction ML Models

For practical application in clinics, the models underwent further refinement through feature engineering based on the quantitative distribution of LC health factors. This refinement led to the development of a concise and practical set of 29 variables, termed “pv29.” Table 3 presents the details of the pv29 variables.

**Table 3:** List of the 29 variables used in the inclusive and practical machine learning models for lung cancer risk prediction.

Category	Local code	Health factor term
Disease	C-572430	Emphysema
Disease	C-654730	Lung inflammation
Disease	C-897420	Bronchitis
History	C-902187	Smoking history
Laboratory test	C-602395	Albumin/globulin ratio
Laboratory test	C-320164	Hematocrit
Laboratory test	C-952408	Non–small cell lung cancer–associated antigen
Laboratory test	C-023789	Carcinoembryonic antigen
Laboratory test	C-945807	Fibrinogen
Laboratory test	C-609483	Lymphocyte ratio
Laboratory test	C-346250	Platelet distribution width
Laboratory test	C-965710	Hemoglobin concentration
Laboratory test	C-546207	Globulin
Laboratory test	C-015328	Alkaline phosphatase
Laboratory test	C-963520	High-sensitivity C-reactive protein
Laboratory test	C-573086	Neuron-specific enolase
Laboratory test	C-284309	Carbohydrate antigen 153
Laboratory test	C-507246	Urine protein
Observation	C-598214	Lung nodules
Observation	C-825049	Pleural effusion
Observation	C-567942	Atelectasis
Risk factor	C-504168	Gender
Risk factor	C-928456	Age
Symptom	C-546879	Cough
Symptom	C-984012	Chest pain
Symptom	C-943817	Shortness of breath
Symptom	C-152064	Coughing up blood
Symptom	C-275809	Chest tightness
Symptom	C-549780	Pain

Table 2 compares the key performance metrics of the base models (XGBoost, RF, SVM, and KNN) using the pv29 variable set with default settings. The pv29 XGBoost and RF models demonstrated comparable performance, achieving 0.80 recall, 0.82 precision, 0.82 AUROC, and 0.82 accuracy. Figure 3 illustrates the ROC and reliability curves of the pv29 XGBoost model. Considering other requirements, including dealing with sparse data in EMRs and compute time, the pv29 XGBoost model was selected as the initial model for the LC risk prediction in initialization of the ML-LHS unit, aimed at the future implementation of risk-based LC-screening recommendations in broader populations.

## Discussion

### Principal Findings

This study introduces a novel ML-LHS unit approach, aiming to offer sustainable and inclusive LC-screening solutions for all at-risk populations in both urban and rural areas within a CRN. To initiate this LC ML-LHS unit, we developed an inclusive and practical XGBoost model for LC risk prediction using hospital EMR data. This enables risk-based LC screening in broader patient populations aged 30 years and older, regardless of smoking status. Using 29 variables, accessible even in low-resource clinics, the ML model achieved LC risk prediction with performance metrics of 0.80 recall, 0.82 precision, 0.82 AUROC, and 0.82 accuracy. Because most of the 29 variables were verified as risk factors or correlated factors for LC in literature, these model outputs are highly plausible. If an end user provides values for the 29 variables to the XGBoost model, the model will return a probability (0%-100%) of LC risk. More than 50% indicates a high risk of having LC, while below 50% indicates a low risk.

### Future Direction: Implementing LC ML-LHS CRN

Considering the challenges in LC screening, such as low-screening adoption and inadequate coverage for nonsmokers and younger patients, exploring risk-based screening strategies is vital [11,33-35]. Following the present study, a future direction involves externally validating the LC risk prediction model. If validated, we plan to deploy the LC ML-LHS unit across a CRN, which will continuously monitor, rebuild the model, validate the new model, and deploy the improved model in so-called “LHS learning cycles.” Once operational, this innovative LHS unit could improve LC-screening rates and early detection in hospitals, community clinics, and rural areas.

Moreover, the ML-LHS CRN is well suited to screen for rare genetic mutations associated with LC, such as the ROS-1 mutation. If certain mutations are identified, personalized and precision medicine may be recommended by a doctor to the patient. Since the pv29 LC model does not contain the genetic mutations as variables, the LHS would need to integrate a large language model (LLM) into the prediction module for treatment prediction task. The top general-purpose LLMs, such as OpenAI’s ChatGPT 4 and Google Gemini 1.5, have shown high accuracy in making medical predictions in our and many other studies without requiring structured data input [36,37]. Enhancing AI applicability through cooperation of structured data ML model and natural language LLMs presents an exciting future research direction.

Furthermore, screening is just the beginning of a patient’s diagnostic journey in an equitable LHS. Future research should also investigate on how AI, particularly generative AI, and LHS can effectively follow up with high-risk patients, educate patients for shared decision-making, and remind patients to underdo diagnostic tests in time for early detection of LC. Simultaneously, LHS will coordinate primary care physicians and specialists to provide the appropriate diagnostics tests, such as image tests (computed tomography, positron emission tomography–computed tomography, and magnetic resonance imaging), pathology tests, and biopsies for final diagnosis. Future studies should also determine when to recommend molecular and genetic testing for achieving personalized and precision treatment.

### Future Direction: Applying the ML-LHS Approach to Other Diseases

The vision of NAM’s LHS emphasizes using RWD to generate real-world evidence. As EMRs are a primary source of RWD, they can be used to develop inclusive and practical ML models for risk predictions of various diseases. Another promising future research direction is applying the ML-LHS unit approach proposed in this study to other preventable diseases and building LHS units in routine health care delivery, aimed at delivering more inclusive predictive screening in underserved populations.

We identify the biggest challenge of applying ML or AI in disease screening for all populations as the difficulty of deployment. ML models requiring a large number of variables may be deployed in hospitals, but they may not be usable in small clinics because the required data cannot be collected there. This study proposes a promising solution to this deployment problem: design a novel ML-enabled LHS unit and strike a balance of minimal variables and acceptable performance for the starting ML model of the LHS. Reducing the number of variables in a practical model usually reduces mode performance compared with the inclusive mode. Setting 80% recall, precision, and accuracy as the acceptance bar, this study of the LC model and previous study of the nasopharyngeal cancer model demonstrated that it is possible to reduce the number of variables to below 30 [27].

For feature engineering, a common method is to use the feature importance list from the ML model. To meet the requirements of reducing variables to a minimal while keeping performance metrics above an acceptable level in starting up an ML-LHS unit, we have proposed an alternative approach that uses a quantitative distribution of health factors generated directly from EMR data by the patient graph CDR method in previous studies [26,27,32]. This study demonstrated again the effectiveness of the new feature selection approach of using health factor distribution from the CDR method in developing inclusive and practical ML models.

### Limitations and Responsible AI

This study, however, has limitations. The EMR data presented issues with bias and missing data [38,39], which could potentially lead to biased models. For instance, smoking status and family history of LC were underreported in our data set. Significant efforts were made to understand and address these data biases, excluding variables where potential bias was identified. Despite these efforts, some biases may remain undetected and unmitigated. We also used algorithms such as XGBoost, known for effectively handling missing data. The lack of standardized structured data in EMRs made data collection labor-intensive. Reducing variables for practicality might risk overfitting in a small data set, though this issue should diminish as the ML-LHS unit continuously accumulates more data through its prediction service [40].

To further address these data bias issues as well as ML or AI application inequities, ML-LHS CRN will emphasize responsible AI development in future research [41]. First, CRN will strive to include more clinics from communities and rural areas surrounding the lead hospital, providing access to a broader population for AI-based LC screening. Second, the ML model will be frequently updated with new data from all patients, particularly including underserved populations, to continuously make the ML data set more representative and less biased. Third, a governance committee should be established to review the development and use of the ML models to ensure high ethical standards, including protection of data safety and patient privacy, minimizing potential bias in data and algorithmic decision-making. Fourth, because mistakes or errors in AI prediction may cause harm or even deadly consequences, AI will be used only as a new information source for medical professionals or patients to make health care decisions.

### Conclusions

This study devised an innovative ML-LHS unit for a CRN to sustainably offer inclusive LC screening to all at-risk populations. For initializing such an ML-LHS unit serving community and rural clinics, we developed an inclusive and practical XGBoost model from hospital EMR data. Future deployment of the LC ML-LHS unit is expected to significantly improve LC-screening rates and early detection in broader populations, including those typically overlooked by existing LC-screening guidelines, such as nonsmokers and younger patients.

## Abbreviations

AI: artificial intelligence
AUROC: area under the receiver operating characteristic curve
CDR: connection delta ratio
CRN: clinical research network
EMR: electronic medical record
KNN: K-nearest neighbors
LC: lung cancer
LHS: learning health system
LLM: large language model
ML: machine learning
ML-LHS: ML-enabled LHS
NAM: US National Academy of Medicine
PDJ: patient diagnosis journey
RF: random forest
ROC: Receiver operating characteristic curve
RWD: real-world data
SVM: support vector machines

## Data Availability

The patient data sets used in the study are not available due to patient data privacy protection. Other data without privacy concern are available from the corresponding authors upon reasonable request.
